# Development of a Real-Time Resistance Measurement for *Vibrio parahaemolyticus* Detection by the Lecithin-Dependent Hemolysin Gene

**DOI:** 10.1371/journal.pone.0072342

**Published:** 2013-08-26

**Authors:** Guiming Xiang, Xiaoyun Pu, Dongneng Jiang, Linlin Liu, Chang Liu, Xiaobo Liu

**Affiliations:** Department of Clinical Laboratory, Xinqiao Hospital, Third Military Medical University, Chongqing, China; University of Houston, United States of America

## Abstract

The marine bacterium *Vibrio parahaemolyticus (V. parahaemolyticus)* causes gastroenteritis in humans via the ingestion of raw or undercooked contaminated seafood, and early diagnosis and prompt treatment are important for the prevention of *V. parahaemolyticus*-related diseases. In this study, a real-time resistance measurement based on loop-mediated isothermal amplification (LAMP), electrochemical ion bonding (Crystal violet and Mg^2+^), real-time monitoring, and derivative analysis was developed. *V. parahaemolyticus* DNA was first amplified by LAMP, and the products (DNA and pyrophosphate) represented two types of negative ions that could combine with a positive dye (Crystal violet) and positive ions (Mg^2+^) to increase the resistance of the reaction liquid. This resistance was measured in real-time using a specially designed resistance electrode, thus permitting the quantitative detection of *V. parahaemolyticus*. The results were obtained in 1–2 hours, with a minimum bacterial density of 10 CFU.mL^−1^ and high levels of accuracy (97%), sensitivity (96.08%), and specificity (97.96%) when compared to cultivation methods. Therefore, this simple and rapid method has a potential application in the detection of *V. parahaemolyticus* on a gene chip or in point-of-care testing.

## Introduction

Although seafood is part of a healthy diet, seafood-associated infections induce a wide variety of clinical syndromes and are caused by a variety of pathogens, including bacteria, viruses, and parasites [1]. *Vibrio parahaemolyticus*, a Gram-negative marine bacterial pathogen, is emerging as a major worldwide cause of food-borne illnesses due to the consumption of raw seafood. *V. parahaemolyticus* infections can lead to such diseases as gastroenteritis, wound infections, and septicemia [2], with the distal small intestine being the major site of *V. parahaemolyticus*-induced tissue damage, reduced epithelial barrier function, and inflammation [3]. The vast majority of environmental *V. parahaemolyticus* isolates are nonvirulent, yet strains of this bacterium remain the leading causes of raw or undercooked seafood-related gastroenteritis [4]. Indeed, each *Vibrio spp.* identified in water and sediment was found to correlate with several environmental measurements, with water temperature and total *Vibrio* level correlating strongly with the occurrence of *Vibrio vulnificus, Vibrio cholerae and Vibrio parahaemolyticus* [5]. Furthermore, as 172 *V. parahaemolyticus* isolates have been identified in patients with diarrhea, freshly harvested sea fish, or freshwater samples from Shanghai, China [6], early diagnosis and prompt treatment are important for the prevention of serious complications such as toxic shock,dehydration and consciousness disorders.

The accurate identification of *V. parahaemolyticus* in samples is very important within the context of public health [7]. Standard microbiological approaches to plate culture and classification, which was generally used and accepted, are unfortunately not useful for control of exposure to pathogenic *V. parahaemolyticus* [8]. Nucleic acid-based molecular methods are a good alternative, as these techniques can accurately differentiate between species and can detect low-level infections [9]. Specific or universal genes, including toxin genes and 16S rRNA genes, have been used in polymerase chain reaction (PCR) assays as target markers to detect different *V*. *parahaemolyticus* strains [10]. In the majority of clinical isolates, randomly amplified polymorphic DNA (RAPD)-PCR produces a unique 600-bp amplicon that was rarely observed in tested environmental isolates [11]. Thermostable direct hemolysin, which is encoded by the *tdh* gene, is considered to be an important virulence factor in pathogenic *V*.*parahaemolyticus* and the targeted amplification of this gene involves a 6-µL reaction volume and an extremely reduced reaction run time, as one cycle can be completed in 10 seconds or less. Consequently, a 35-cycle ultra rapid real-time PCR can successfully detect up to 100 fg (18 copies) of *V. parahaemolyticus* [12].

Loop-mediated isothermal amplification (LAMP) is a simple method that can be improved for use in endemic countries [13]; indeed, LAMP use has already advanced the detection of viruses, bacteria, and parasites and food safety analysis. Previously, Wang et al. developed a reverse transcription LAMP (RT-LAMP) assay for the detection of porcine teschovirus [14]. A significant difference in *V. parahaemolyticus* detection was observed between real-time PCR and LAMP assays. Estimates of detection accuracy of total *V. parahaemolyticus* by latent class analysis showed <90% statistical sensitivity for the LAMP assay, regardless of template utilized, indicating greater false negative reporting than the other PCR methods with statistical sensitivities of 92–97%. But all methods demonstrated a statistical specificity of 94% or greater, indicating little to no false positive reporting by LAMP or real-time PCR assay [15].

Many methods have been developed on gene chips or for point-of-care testing(POCT), including pyrolysis, template synthesis, hydrothermal synthesis, microemulsion, and electrochemical methods. Among these, the electrochemical methods are favored due to their relatively good controllability, ease of operation, and mild reaction conditions. By detecting the voltage, current, resistance, and other relevant signal using different kinds of electrode, substance concentration can be electrochemically measured accurately and quickly [16].

**Figure 1:**
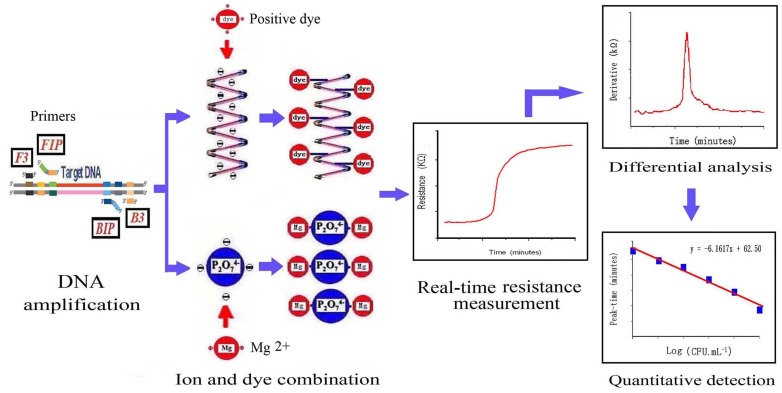
Scheme of the real-time resistance measurement for *V. parahaemolyticus*. Briefly, the lecithin-dependent hemolysin (LDH) gene of *V. parahaemolyticus* was amplified by LAMP at first. The subsequent two products, DNA and pyrophosphate, both negative ions, were combined with a positive dye (Crystal violet) and positive ions (Mg2+), leading to an increase in the reaction liquid resistance. This resistance was measured in real-time using a electrode, and *V. parahaemolyticus* concentration was quantitatively detected through a derivative analysis.

In this study, real-time resistance measurement [17], a LAMP-based electrochemical method was developed to detect *V. parahaemolyticus* in patient’s faces. The purpose of this study was to develop an accurate, quick DNA analysis method and prove the superior capacity of the molecular technique to detect *V. parahaemolyticus* DNA. The schematic diagram of this experiment is shown in Figure 1.

## Materials and Methods

### 1. Reagents and Equipments

The DNA amplification kits (DNA-LAMP) including positive and negative controls were purchased from Eiken (Japan), a constant concentration of magnesium (6.0 mg/L) had been included in the kits. The total bacterial DNA extraction kits were provided by Tiangeng (China). Crystal violet was obtained from Sigma-Aldrich (USA) and millipore filter of 1.2 µm pore size was from Millipore(USA). The bacterial media were produced by Pangtong (China), and WGZ-2-XJ bacterial transmissometer was produced by Xinrui(China). The API 20E identification cards were provided by Bio-Merieux (France). The platinum(Pt) electrode and argentum/argentum chloride(Ag/AgCl) electrode were purchased from Dropsens (Spain). The VICTOR 86C digital multimeter was produced by Victor(China), and the ALB64 thermobath was produced by Finepcr (Korea).

### 2. Bacterial Operation

Standard bacterial strains were obtained from American Type Culture Collection (ATCC, USA), including *V. parahaemolyticus* (ATCC17802) and 13 additional bacterial strains: *Clostridium perfringens* (ATCC13124), *Clostridium difficile* (ATCC9689), *Clostridium tetani* (ATCC19406), *Clostridium histolyticum* (ATCC19401), *Clostridium septicum* (ATCC12464), *Acinetobacter baumannii* (ATCC19606), *Enterococcus faecalis* (ATCC14506), *Haemophilus influenzae* (ATCC 10211), *Escherichia coli* (ATCC25922), *Staphylococcus aureus* (ATCC25923), *Pseudomonas aeruginosa* (ATCC27853), *Streptococcus pneumoniae* (ATCC49619), and *Neisseria gonorrhoeae* (ATCC19424). All standard bacterial strains were stored at −70°C and cultured different selective on agar medium before used. Fresh feces specimens were plated on *V. parahaemolyticus* agar medium directly and single bacterial colony was used for identification after a 24-h incubation at 37°C. Bacterial identification was performed with API 20E identification cards according to the manufacturer’s recommended protocol.

### 3. Primer Design and Synthesis

Thermolabile hemolysin encoded by *V. parahaemolyticus* lecithin-dependent hemolysin (LDH) gene had the specialty, not only environment isolated strains but also clinical isolated strains have the gene. Nucleotide sequences of LDH gene were retrieved from the National Center for Biotechnology Information (NCBI) and used as target DNA. LAMP primers including forward primer(F3), reverse primer(B3), forward inner primer (FIP), and reverse inner primer(BIP) were designed to correspond to conserved regions using Primer Explorer 4.0 online software (Eiken, Japan) and were synthesized by Sangong (Shanghai, China).

### 4. Real-time Resistance Measurement

Bacterial solution were prepared by dissolving one single bacterial colony or 0.1 g patient’s fresh feces in 5 ml sterile phosphate-buffered saline. After filtered by 1.2 µm millipore filter all bacterial solution were centrifuged at 2000×g for 5 min and the resulting 500 µL precipitation was used for DNA extraction. Template DNA was alkaline extracted according to the manufacturer’s recommended protocol and stored at −20°C prior to use. The LAMP reactions were performed in PCR reaction tubes with 2 µL DNA extract, 12.5 µL reaction reagent, 1.0 µL Bst DNA polymerase, 4 µL primer mixture (containing 4 primers: 10 µmol.L^−1^ F3 and B3 and 40 µmol.L^−1^ FIP and BIP), and 5.5 µL double-distilled water. The 25 µL mixtures were incubated at 65°C for 60 minutes in an ALB64 thermobath. A four-trode Pt electrode was designed for real-time resistance monitoring and to avoid the inhibition of LAMP. The electrode surface was electroplated with gold nanoparticle to increase electrochemical stability in the reactions. The real-time electrochemical measurements were performed using the VICTOR 86C digital multimeter and an Ag/AgCl electrode was used as a reference. A derivative analysis of the test results was performed with OriginPro 7.5 software (OriginLab, USA).

### 5. Specificity Analysis

*V. parahaemolyticus* and the 13 other common strains of bacteria were cultured on different selective agar medium by a 24-h incubation at 37°C respectively. Bacterial solution was prepared by sterile phosphate-buffered saline using single bacterial colony and diluted to the same concentration (1×10^6 ^CFU.mL^−1^) on WGZ-2-XJ bacterial transmissometer. The total bacterial DNA was extracted and real-time resistance was measured as described above.

### 6. Sensitivity and Regression Analysis

*V. parahaemolyticus* was grown on *V. parahaemolyticus* agar medium. Counts were performed by plating 100-µL dilutions in sterile phosphate-buffered saline onto agar plates, followed by a 24-h incubation at 37°C. The average number of colony forming units (CFU) was calculated post-incubation. Additionally, a solution of *V. parahaemolyticus* was serially diluted (from 1×10^6^ to 1×10^1 ^CFU.mL^−1^); DNA was extracted from the different dilutions, and *V. parahaemolyticus* concentration was quantitated by CFU. The derivative peak times from the real-time resistance measurement were calculated using OriginPro 7.5 software. Three samples of the same bacterial concentration were measured twice and all values were recorded as mean(n = 6). The semi-logarithmic linear regression was analyzed with Excel 2003 (Microsoft, USA).

### 7. Comparative Analysis of the Real-time Resistance Measurement with Culture Identification

A total of 100 feces specimens were collected by us from different raw seafood-related gastroenteritis patients in our hospital. All patients had a raw seafood-contacted history recently. parallel detected by the real-time resistance measurement and the culture identification within two hours respectively. Positive means the increase of resistance was at least twice higher than the negative control or at least one *V. parahaemolyticus* colony was isolated and identified in each specimen. Culture identification was used as the reference method and the statistical significance of differences in agreement between methods was determined by Chi square test. p<0.05 represents the statistical difference between the two methods was significant.

The protocol for collecting feces samples was approved by the Ethics Committee of the Xinqiao Hospital, Chongqing.

## Results

### 1. Primer Design

The *V. parahaemolyticus* LDH gene (GeneBank: BA000032.2) was selected for primer design by exploring the NCBI GeneBank database. The specificity of primer sequence was positively tested by basic local alignment search tool (BLAST)on NCBI website (Table 1).

**Table 1:** Primers for *V. parahaemolyticus.*

Lecithin-dependent hemolysin (LDH) gene. (GeneBank: BA000032.2)
Primers (5′-3′):Forward primer(F3): CACCAGTAGCCGTCAATGReverse primer(B3): TAACTGCATTACTCCCGCForward inner primer (FIP): TATCGCACCAGCTACTCGAAAGCATCTTCGTTTTTTGCCCATReverse inner primer(BIP):ACAGCGAACATAGGTATAGGTTTGGAGAGCCAACCTTATCACC

### 2. Specificity Analysis

**Figure 2:**
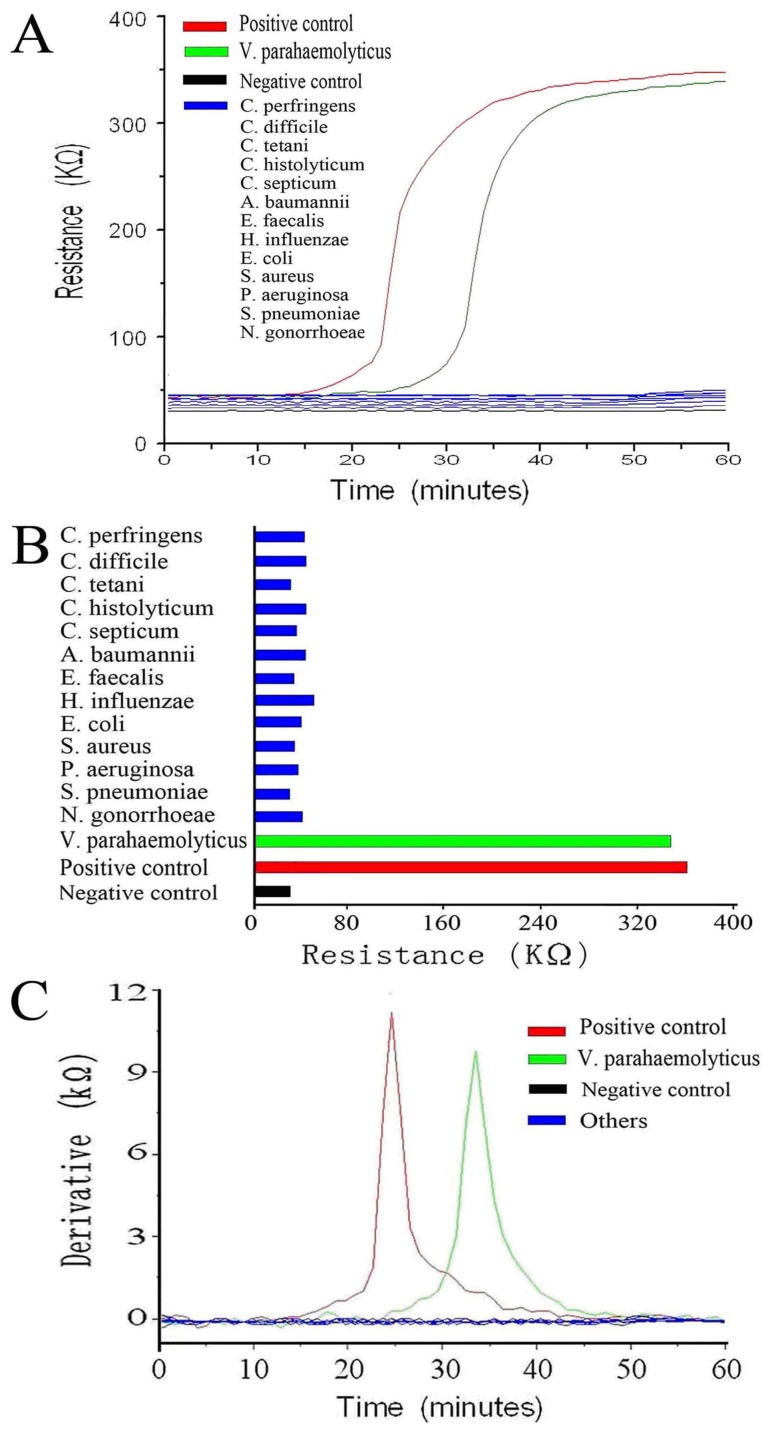
Specificity analysis of the real-time resistance measurement. A. The real-time resistance curve of *V. parahaemolyticus* and other interfering bacteria. B. End-point resistance of *V. parahaemolyticus* and other interfering bacteria after a 60-min LAMP assay. C. Derivative analysis of the real-time resistance measurement.

*V. parahaemolyticus* was positively detected by our real-time resistance measurement, whereas the other 13 kinds of bacteria were not detected (Figure 2A and B), suggesting that the LAMP assay is highly specific for the detection of *V. parahaemolyticus*. We observed the same results in a derivative analysis of the measurements (Figure 2C); the results of the derivative analysis were more clear, and it was easier to quantitatively detect *V. parahaemolyticus* using this method.

### 3. Sensitivity and Regression Analysis

**Figure 3:**
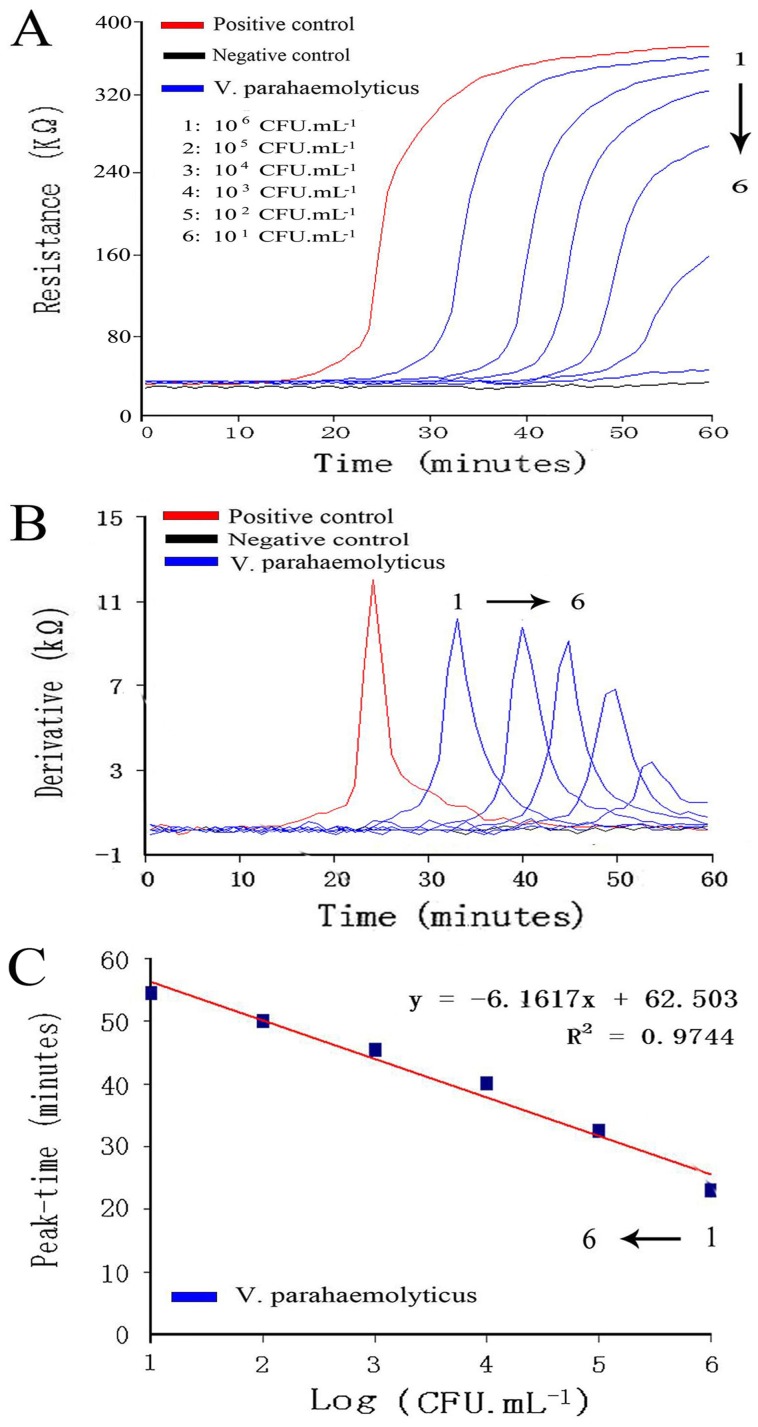
Sensitivity and regression analyses of the real-time resistance measurement. A. The real-time resistance curve of *V. parahaemolyticus* in a concentration gradient. B. The derivative analysis of the real-time resistance measurement of *V. parahaemolyticus*. C. The regression analysis of the real-time resistance measurement of *V. parahaemolyticus.* Three samples of the same bacterial concentration were measured twice and all values were recorded as mean(n = 6).

An analysis of the real-time resistance measurement indicated that the *V. parahaemolyticus* detection limit was 1×10 CFU.mL^−1^ according to a signal to noise ratio of 2. (Figure 3A). This result indicates a high sensitivity. Additionally, the real-time resistance measurement performed well for the derivative calculation (Figure 3B). It was obvious that a linear relationship was existed between the *V. parahaemolyticus* concentration and the differential peak-times. The linear range covered from 1×10^2^ to 1×10^6^ CFU.mL^−1^ with a regression equation of the form y = −6.1617x+62.503 and a linear correlation coefficient of 0.9744 (Figure 3C). Thus, the *V. parahaemolyticus* concentrations in the samples could be quantitatively extrapolated by derivative analysis.

### 4. Comparison of Real-time Resistance Measurement with Culture Identification

The results of the X^2^ test shown in Table 2 suggested a lack of significant differences between the real-time resistance measurements and culture identification(P = 0.564). Additionally, the accuracy, sensitivity and specificity of our measurement were 97%, 96.08% and 97.96% respectively compared to the standard cultivation method.

**Table 2:** Comparison of real-time resistance measurement and culture identification.

		culture identified		X2 test	
		V. parahaemolyticus	Other bacteria	X2	P
Real-time resistance measured	+	48	1	0.333	0.564*
	−	2	49		

* There is no statistical difference between the two methods (P>0.05).

## Discussion

*V. parahaemolyticus* is a leading cause of seafood-borne gastroenteritis in many parts of the world. The distal small intestine is the major site of *V. parahaemolyticus*-induced tissue damage, reduced epithelial barrier function, and inflammation, suggesting that disease in this region of the gastrointestinal tract primarily accounts for the diarrhea associated with *V. parahaemolyticus* infections. Therefore, early diagnosis and prompt treatment are important to *V. parahaemolyticus* infection disease.

Bacterial identification via cultivation is currently considered a “gold standard” technique in clinical laboratory. However, cultivation requires relatively high-standard laboratory facilities and is time-consuming, thus leading to delays beyond the optimal time window for treatment. Nucleic acid-based molecular methods are a good alternative, as these techniques can accurately differentiate between species and can detect low-level infections. Unfortunately, high nucleotide similarity often exists among genes from different bacterial species, particularly those within the same genus, and the absence of toxin genes in nonvirulent strains has prevented the use of toxin genes as targets for species-specific bacterial pathogen identification [18]. The use of high annealing temperatures reduces the overall PCR duration by at least 1 h, permitting the rapid detection of *V. parahaemolyticus* in the fish tissues, which might be due to the presence of inhibitory materials. The primer detection limits were reported to be 100 pg and 1 ng for DNA purified from bacterial culture and infected oyster tissue, respectively [19], and a previous study reported that PCR-ELISA increased the detection sensitivity for *V. parahaemolyticus* by 100-fold in comparison to gel-based detection methods [20]. Electrochemical methods have attracted considerable interest because of its intrinsic advantages such as portability, low cost, high sensitivity, and low power requirement. In this study, we reported a modified method of DNA detection based on a combination of techniques, including LAMP and electrochemical binding.

In our real-time resistance measurement the electrochemical detection of *V. parahaemolyticus* could be completed in 1–2 hours, with a low detection limit of 10 CFU.mL^−1^. It showed that the sensitivity of the real-time PCR assay described above was slightly higher than that of our method. But the real-time PCR assay often requires expensive equipment and reagents. Compared to the other LAMP or real-time PCR methods with sensitivities of 87–97% and specificity of 94–100%, the sensitivity and specificity of our method were acceptable [15]. Moreover, the essential equipment was cheap and the measurement process was simple. Additionally, it was suitable for the *V. parahaemolyticus* detection in real feces sample. However, the utilization of the real-time resistance measurement was minimized due to the limited supply of the LAMP DNA polymerase. The potential cross-contamination also should be noted when repeat using a electrode.

In summary, the real-time resistance measurement is a simple and rapid *V. parahaemolyticus* detection method with a high sensitivity and specificity. This method had promising analytical applications in the detection of *V. parahaemolyticus* on gene chips or in POCT.
